# Microecological treatment of hyperuricemia using *Lactobacillus* from pickles

**DOI:** 10.1186/s12866-020-01874-9

**Published:** 2020-07-06

**Authors:** Yuanxun Xiao, Congxin Zhang, Xianli Zeng, Zhichao Yuan

**Affiliations:** Department of LiWan Hospital, Third Affiliated Hospital of Guangzhou Medical University, Guangzhou, 510150 Guangdong China

**Keywords:** Lactic acid bacteria, Pickles, Hyperuricemia, Microecology, Uric acid

## Abstract

**Background:**

Hyperuricemia is one of the important risk factors for gout, arteriosclerosis, cardiovascular and cerebrovascular disease. *Lactobacillus* has attracted much attention due to its role in the regulation of intestinal function and tumor resistance, but its ability to reduce uric acid is unclear. Pickles are a traditional fermented food rich in lactic acid bacteria (LAB).

**Results:**

LAB strains were isolated from 18 pickles and their tolerance to acid bile salts, trypsin, pepsin were evaluated after screening by nucleoside degradation. 16S rDNA sequence analysis was used to identify LAB strains. Furthermore, we established rat model of hyperuricemia and demonstrated that *Lactobacillus* could alleviate hyperuricemia and reduce kidney injury.

**Conclusion:**

This study suggests that microecological treatment with *Lactobacillus* represents a feasible option for patients with chronic hyperuricemia.

## Background

Hyperuricemia is a progressive chronic disease without obvious clinical symptoms [1]. Abnormal uric acid in the blood can induce gout, and is an important risk factor for arteriosclerosis, cardiovascular and cerebrovascular disease [2–4]. It is known that Western diet is rich in purine and adenine. In addition, the purine compounds disodium 59-guanylate and disodium 59-inosinate are the main components of many flavor enhancers that are widely used in modern food production [5]. At present, several clinically available drugs can control acute gout, but most patients with hyperuricemia are unable to achieve long-term control due to high purine diets and poor treatment efficacy [6]. For example, allopurinol can induce hypersensitivity syndrome, which may lead to the death of patients [7]. Therefore, the use of purine compound degrading probiotics is a promising alternative for the prevention of hyperuricemia.

Lactic acid bacteria (LAB) are Gram positive, acid-tolerant, fermenting rods or cocci that produce lactic acid as the major metabolic end-product of carbohydrate fermentation [8]. In recent years, *Lactobacillus* has gained interest due to the ability to regulate molecular microbial ecology in the human body [9]. Some LAB strain could reduce serum uric acid level by assimilating purine nucleosides because it produced inosine hydrolase which degraded inosine in vitro, and reversed high-fructose induced increase of uric acid by reducing inosine [10–12].

Pickles are traditional fermented food that produce abundant LAB in a short timeframe under sealed air and acidic conditions. However, whether LAB from traditional pickles can reduce uric acid levels remains unclear. In China, it is still not widely accepted to take daily supplement of probiotics such as LAB, while most Chinese eat pickles due to the low price and easy accessibility. Therefore, in this study we aimed to investigate the potential of *Lactobacillus* from pickles as a new microecological approach for the clinical treatment of hyperuricemia. We established rat model of hyperuricemia and examined whether *Lactobacillus* could alleviate hyperuricemia and reduce kidney injury in the rats.

## Results

### Isolation of LAB strains

**Fig. 1:**
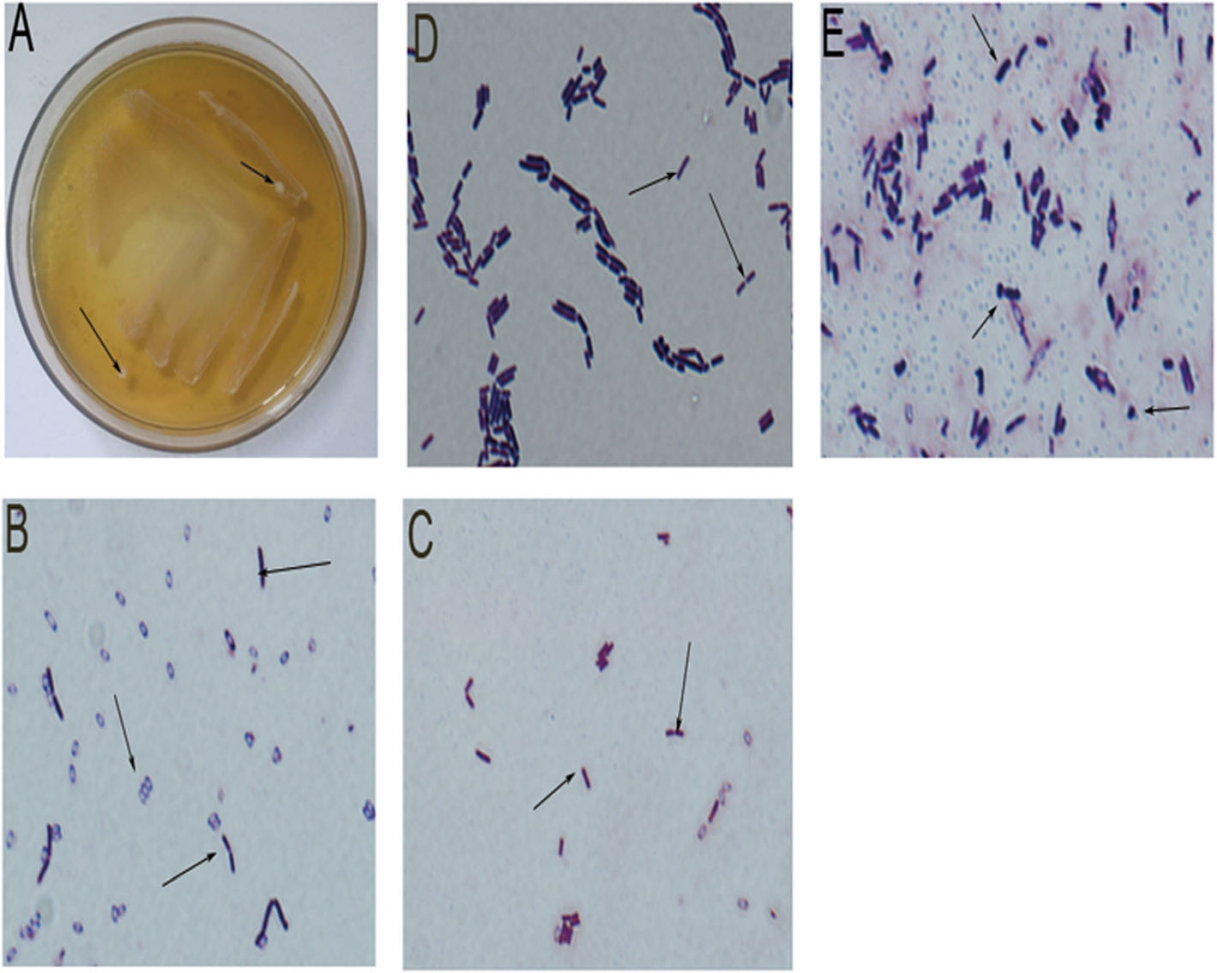
Isolation of LAB strains. **a**. MRS culture plate. **b**. S1 strains showed Rhabditi form under high-power microscope (100× oil). **c**. S5 strains showed spherality form under high-power microscope (100× oil). **d**. S12 strains showed Rhabditi form under high-power microscope (100× oil). **e**. S14 strains showed Rhabditi form observed under high-power microscope (100× oil)

LAB strains were isolated from most pickles, but large differences in the colonies across the pickles were observed (Table 1). Fourteen strains from 18 different pickles were screened as gram staining positive and H_2_O_2_ test negative (Table 2). LAB were predominantly rod shaped and globular (Fig. 1b-e). We also observed colonies containing various LAB types (Fig. 1b-e).

**Table 1:** The number of LAB colonies isolated from pickles

Number	Product	Origin	Total bacterial colony ^a^	*Lactobacillus* colony ^b^
1	Lactobacillus Yogury	GuangZhou, GuangDong	2	1
2	Erie milk	HuErHaoTe, Inner Mongolia	1	1
3	Lllie Lactobacillus	HuErHaoTe, Inner Mongolia	18	1
4	Ferment Powder (lacto)	HaiDian, BeiJing	1	1
5	Stewed Bamboos	YongFeng, JiangXi	6	0
6	Sour Beans	ChengDu,SiChuan	25	0
7	Kraut	MeiShan, SiChuan	18	2
8	Pickle (vegetables)	ChengDu,SiChuan	65	1
9	Pickle (vegetables)	BaoShan, YunNan	59	1
10	Fish Sauerkrant	WeiYan,SiChuan	9	1
11	Pickle (lotus root)	JinZhou, HuBei	24	1
12	Pickle (cabbage)	XinMin,ShenYang	80	1
13	Pickle (vegetables)	XinDu, ChengDu	7	1
14	Pickle (vegetables)	ShenYang,LiaoNing	22	1
15	Pickle (vegetables)	QingZhen, GuiZhou	36	1
16	Pickle (lettuce)	YueYang, HuNan	71	0
17	Pickle (kelp)	YueYang, HuNan	32	0
18	Pickle (cabbage)	ChengDu, SiChuan	6	1

Note: a. Total number of bacterial colony grown on MRS medium. b. Total number of LAB colony grown on MRS medium

**Table 2:** The characteristics of different LAB strains

Strain	Source	Form	24 h pH ^a^	H_2_O_2_ test ^b^	Gram stain ^c^
S1	Lactobacillus Yogury	Rhabditi form	6.31–3.86	–	+
S2	Erie milk	Rhabditi form	6.33–3.89	–	+
S3	Lllie Lactobacillus	Rhabditi form	6.33–3.84	–	+
S4	Kraut	Rha/Sph	6.30–3.93	–	+
S5	Kraut	Spherality	6.32–3.75	–	+
S6	Pickle (vegetables)	Rha/Sph	6.32–3.85	–	+
S7	Pickle (vegetables)	Rhabditi form	6.29–3.81	–	+
S8	Fish Sauerkrant	Rhabditi form	6.31–3.91	–	+
S9	Pickle (lotus root)	Rhabditi form	6.32–3.84	–	+
S10	Pickle (cabbage)	Rhabditi form	6.31–3.83	–	+
S11	Pickle (vegetables)	Rhabditi form	6.32–3.84	–	+
S12	Pickle (vegetables)	Rhabditi form	6.31–3.80	–	+
S13	Pickle (vegetables)	Rhabditi form	6.31–3.81	–	+
S14	Pickle (cabbage)	Rhabditi form	6.29–3.79	–	+

Note: a. 24 h change of pH. b. H2O2 test determined the presence of catalase, an enzyme that degrades H2O2. Negative (−) indicated the absence of catalase. c. Gram staining positive (+) indicated the bacteria had thick cell wall. Most LAB could produce acid, lack catalase and are Gram^+^

### Inosine and guanosine degradation by LAB strains

**Fig. 2:**
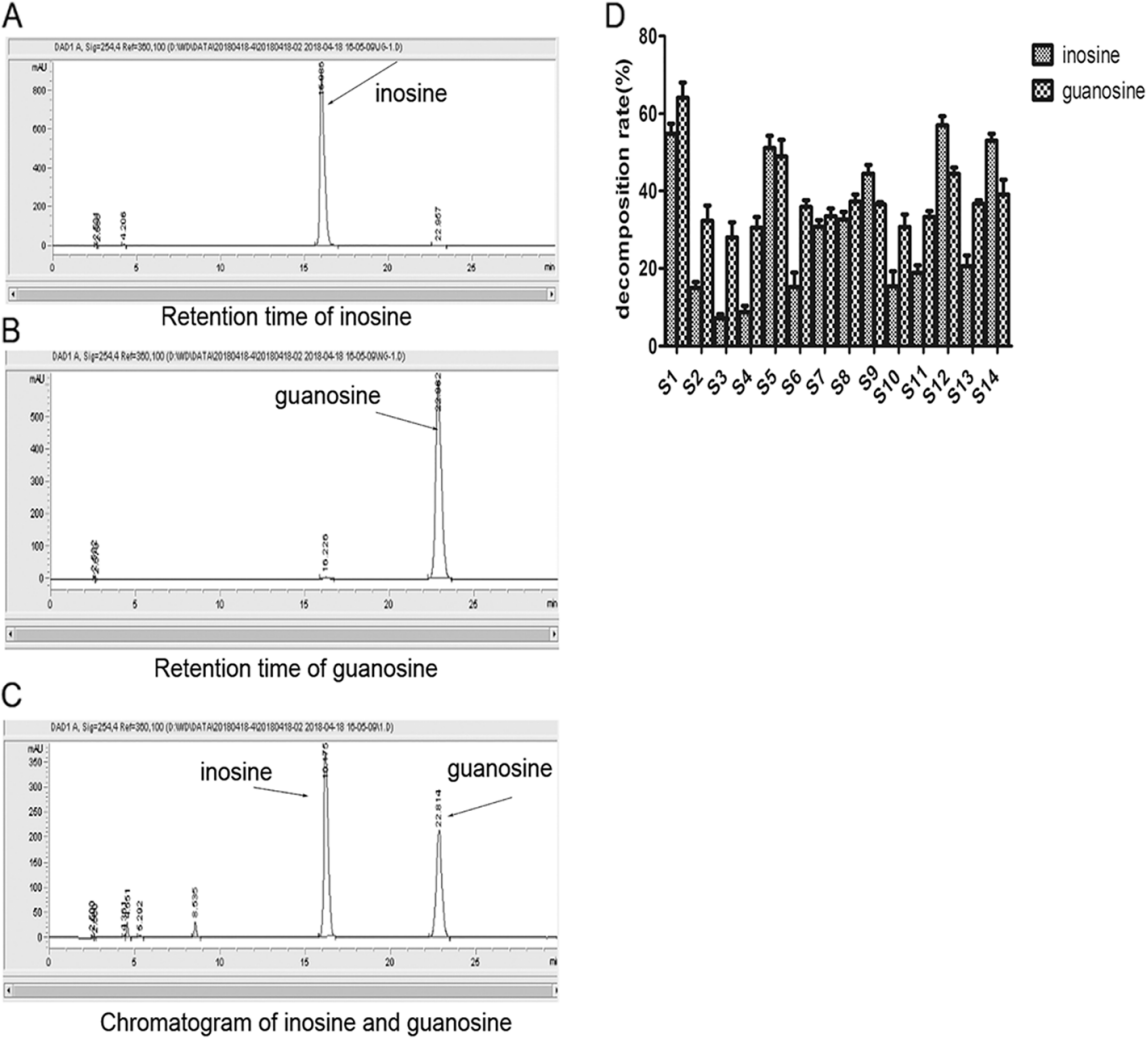
The degradation of inosine and guanosine by LAB strains. **a**. The retention time of inosine in HPLC chromatogram. **b**. The retention time of guanosine in HPLC chromatogram. **c**. The retention time of inosine and guanosine in HPLC chromatogram. **d**. The assimilating speeds of inosine and guanosine showed variations in LAB S1-S14 strains

HPLC chromatograms showed that peak areas of inosine and guanosine were 17,547.2 and 17,522.1, respectively (Fig. 2a, b). Due to favorable discrimination, we were able to distinguish inosine and guanosine (Fig. 2c). We identified 14 strains with various ability to degrade nucleosides. Among the strains, S1, S5, S12 and S14 degraded more than 40% of nucleosides, and were thus selected for further analysis (Fig. 2d).

### Characterization of candidate strains

**Fig. 3:**
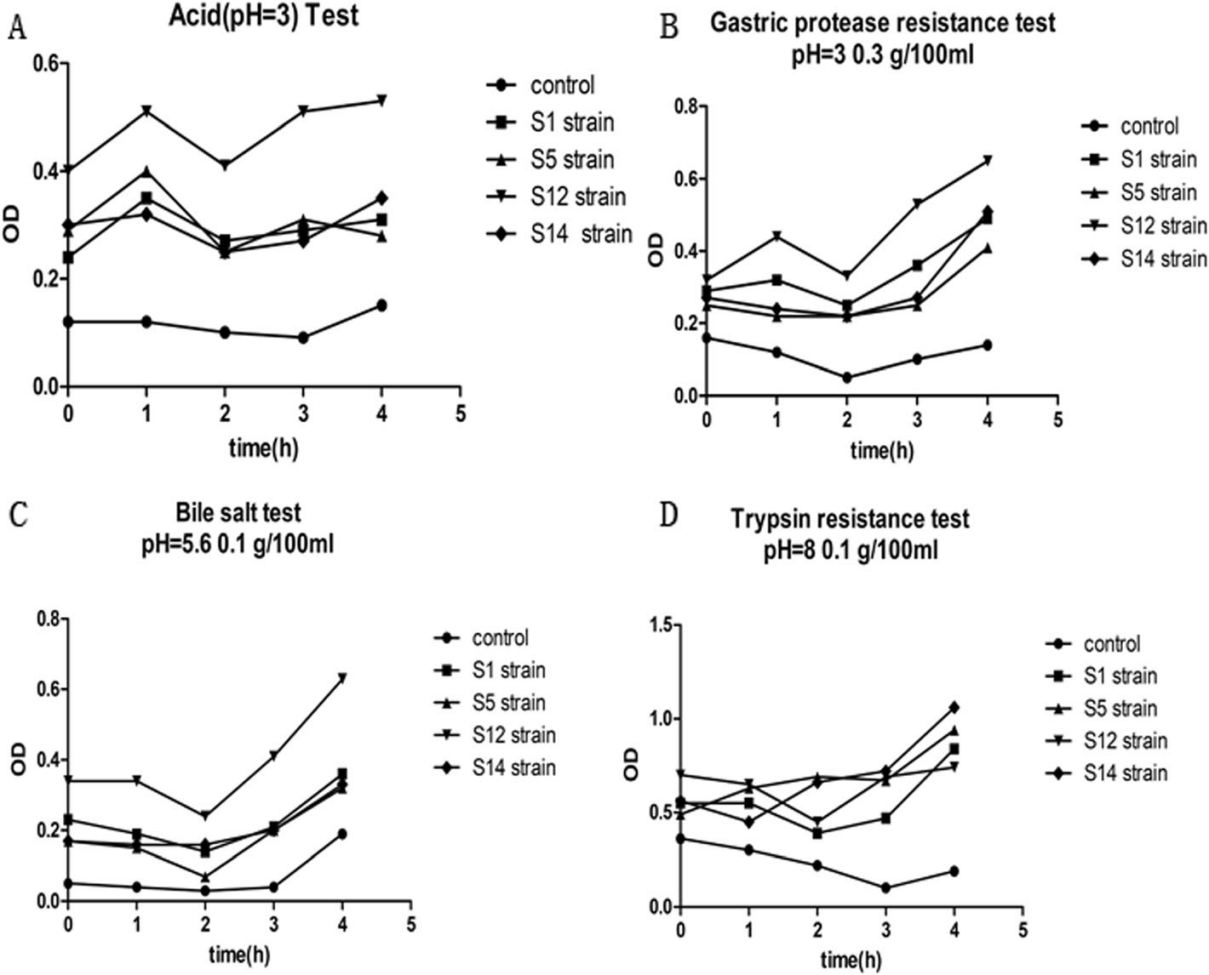
The growth of strains in different conditions. Different strains (S1, S5, S12 and S14) were cultured in liquid MRS medium in the presence of different substances, and OD values were measured over time. **a**. The growth of strains in acid-containing medium. **b**. The growth of strains in gastric protease-containing medium. **c**. The growth of strains in gastric bile-containing medium. **d**. The growth of strains in gastric trypsin-containing medium. Control: liquid MRS only

S1, S5, S12, S14 strains had improved tolerance to acid, pepsin, bile salts and trypsin as the OD values for these strains increased under the treatment conditions. In particular, S12 strain had the highest tolerance among the four strains (Fig. 3a-d). Notably, we found irregularity in OD in control group although we repeated experiments, and because we used liquid MRS medium as control, the pattern of OD change may be due to the reaction of liquid MRS with trypsin over time.

### Identification of candidate strains

**Fig. 4:**
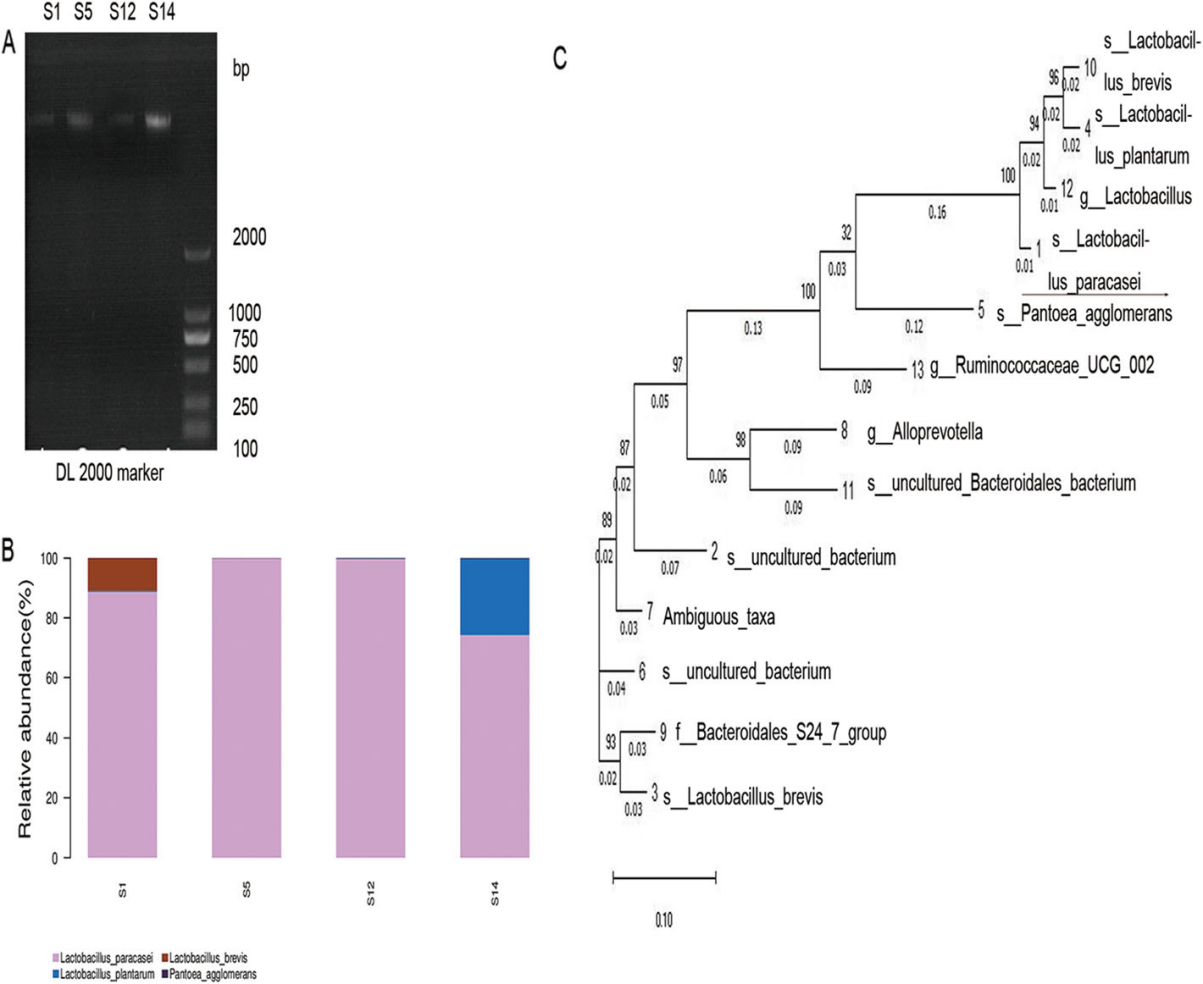
Phylogenetic tree of S1, S5, S12 and S14 strains based on 16S rDNA sequences. **a**. PCR results. **b**. The ratio of each strain in the samples. **c**. The phylogenetic tree based on the 16S rDNA sequences. Reference sequences were obtained from the GenBank nucleotide sequence database (NCBI), including KR066436.1, EF097420.1.1396, DQ824024.1, KX519704.1, GQ853416.1, DQ014606.1, EU791061.1, EF096774.1, AB934477.1, GU295951.1, AB702765.1, DI343235.1, DQ808601.1

**Fig. 5:**
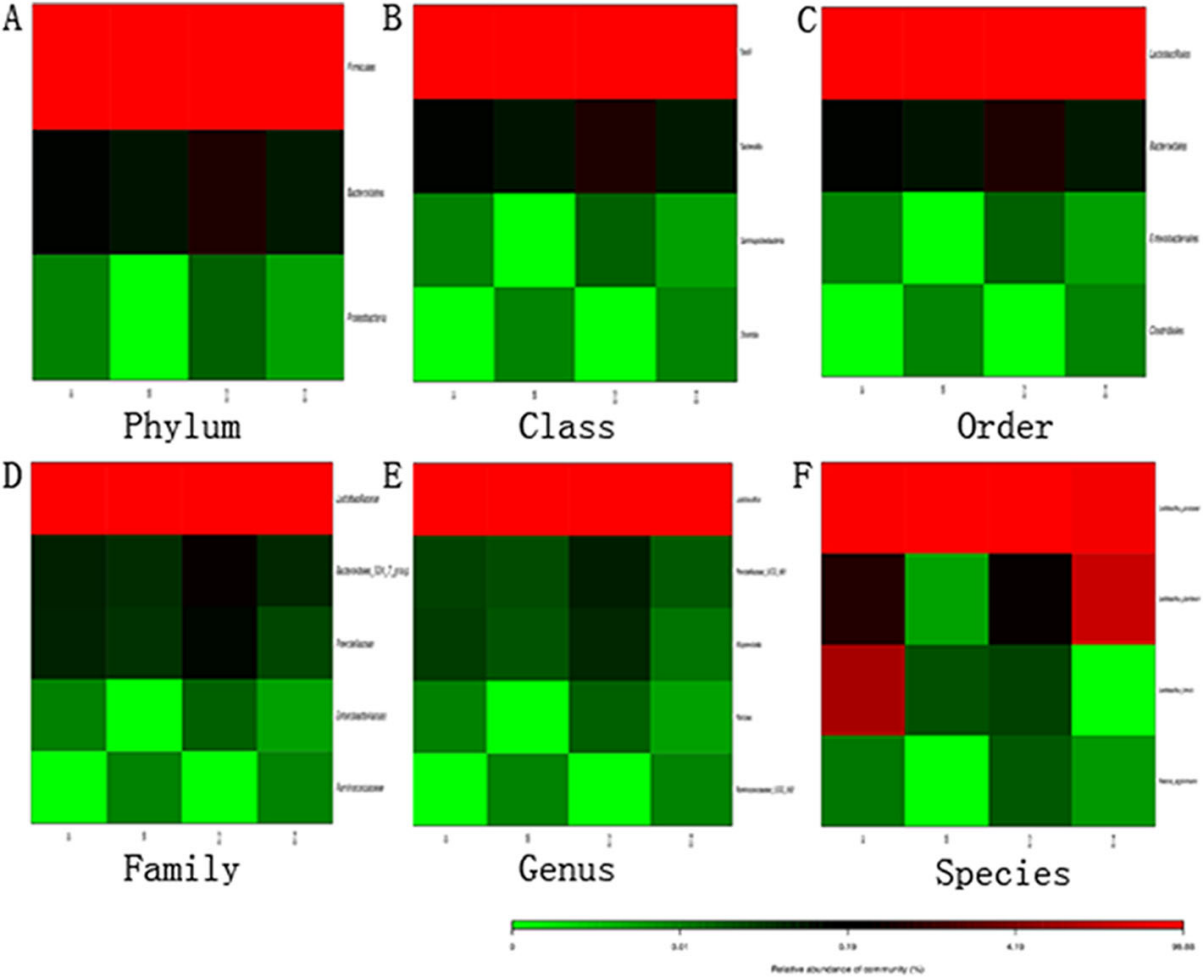
Different abundance of colonies in the Heatmap. **a**. Top 30 of the sample abundance in phylum level. **b**. Top 30 of the sample abundance in class level. **c**. Top 30 of the sample abundance in order level. **d**. Top 30 of the sample abundance in family level. **e**. Top 30 of the sample abundance in genus level. **f**. Top 30 of the sample abundance in species level

Four candidate strains possessed different types of LAB. More than 75% of the four strains were *Lactobacillus paracasei* among which S12 strain had a content as high as 99%. Other LAB present included *Lactobacillus plantarum*, *Lactobacillus brevis and Pantoea agglomerans* (Fig. 4a, b). Compared to the Silva database (v123), the four candidate strains were the same type of *Lactobacillus paracasei* (over 97% homology. (Fig. 4c). We selected S12 strain for further analysis because of the similarity. We also found that pickles included other strains in the different Heatmap level. Firmicutes, Bacteroidetes and Proteobacteria were shown in the phylum levels, and the Firmicutes were over 95% (Fig. 5a). The heatmap showed that several microbial colonies coexisted, but *Lactobacillus* was dominant bacterial community (Fig. 5b-f).

### S12 strain alleviates hyperuricemia in rats

**Fig. 6:**
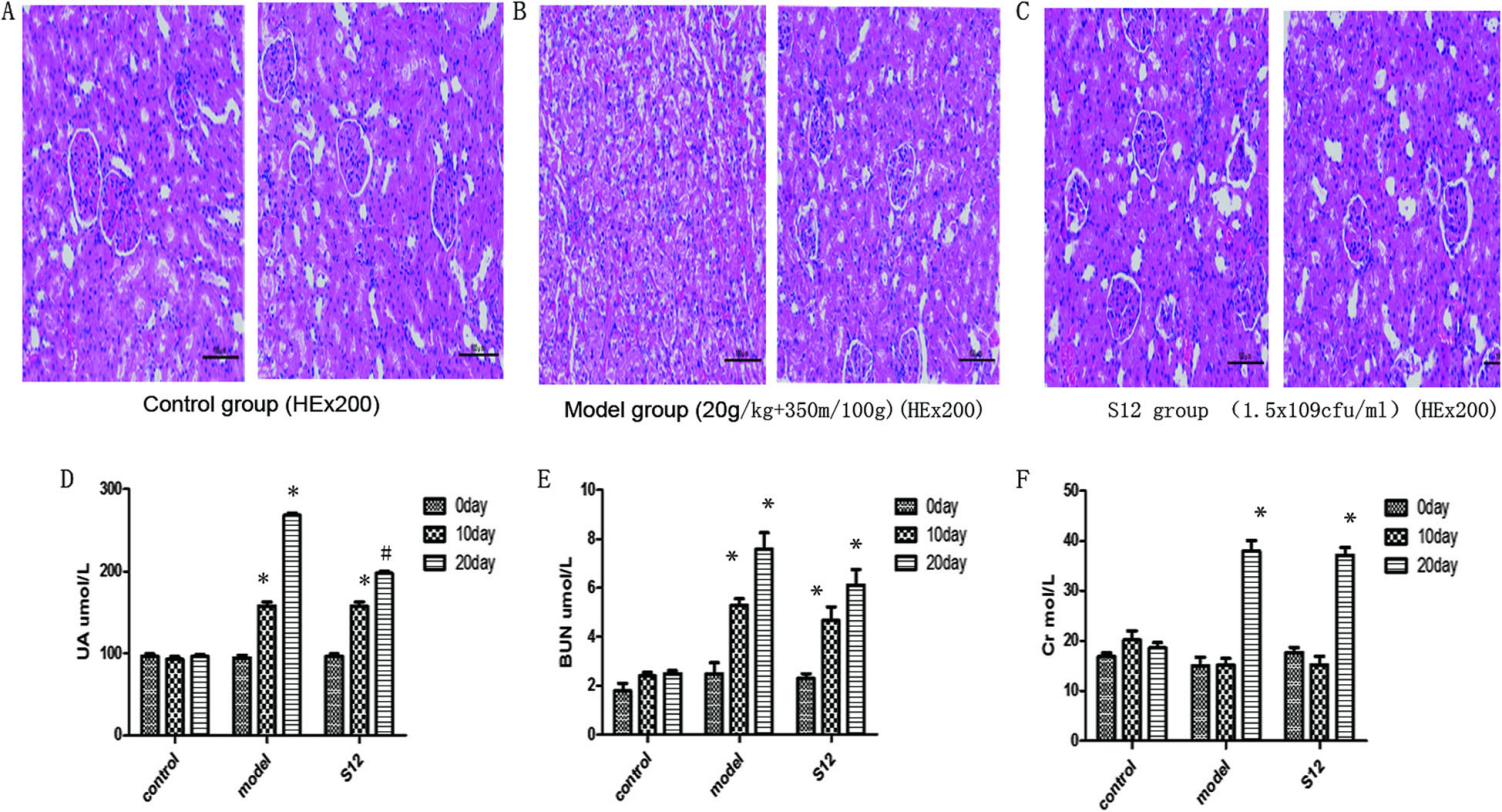
S12 strain alleviates hyperuricemia in rats. **a, b, c**. Histological analysis of kidney tissue sections on day 20 in different rat groups. **d**. The levels of serum UA in different rat groups (*n* = 5). *P < 0.05 compared with control group. #*P* < 0.05 compared with model group. **e**. The levels of serum BUN in different rat groups (*n* = 5). *P < 0.05 compared with control group. **f**. The levels of serum Cr in different rat groups (*n* = 5). *P < 0.05 compared with control group

Upon kidney assessment, we found that the kidneys of S12 group were only partially damaged compared to model group. Renal tubular epithelial cells displayed edema and degeneration in S12 group, but renal glomerulus and tubule structures were still visible. In contrast, model group displayed significant renal damage, including edema and degeneration of the edulla and cortex, and capillary congestion (Fig. 6a-c). Serum UA and BUN of each model group significantly differed from control group on day 10 (*P* < 0.05). Thus hyperuricemia model was successfully established under our conditions. On day 20, serum UA levels in S12 group were lower compared to model group (*P* < 0.05), but serum Cr and BUN increased in all groups compared to control group (*P* < 0.05). There were no changes in serum Cr or BUN levels in control group at the various time points (Fig. 6d-f).

## Discussion

Purine rich foods such as meat and seafood as well as alcoholic beverages potently exacerbate hyperuricemia. Probiotics with the ability to degrade purine compounds in food have been investigated for the prevention of hyperuricemia [10–13]. Pickles are typical representative of Chinese traditional fermented food as a rich natural source of LAB [14]. Microorganisms degrade nucleosides mainly through the biosynthesis of nucleoside hydrolases. Nucleoside hydrolases break the glycoside bonds of nucleosides and release nitrogenous bases, which are further processed by xanthine dehydrogenase to produce uric acid [15, 16]. Thus the existence of nucleoside hydrolase in the strain can be proven by the degradation of nucleoside substances such as guanosine and inosine.

To determine whether the tested strains can survive in the gastrointestinal environment and colonize successfully, the biological characteristics of the four candidate strains were evaluated. The gastrointestinal tract is rich in digestive enzymes, with strong acidic condition to inhibit bacterial growth [11]. Generally, it takes 4 h for food to enter the intestinal tract from the mouth for digestion and absorption. All four strains were well tolerated in the simulated GI environment [11]. We found *Lactobacillus paracasei* to be the representative strain isolated from traditional pickles by 16S rDNA sequence analysis [17]. S12 strain had the highest similarity of *Lactobacillus paracasei* (99.33%), and was used for in vivo animal experiments. In addition, we investigated the taxonomy of microbial colonies in pickles, including Firmicutes, Bacteroidetes and Proteobacteria. These data help better understanding of the microbial environment of pickles.

We established a hyperuricemia model in rat by combining yeast-extract with oxonic acid potassium [18]. However, it has been difficult to establish animal hyperuricemia model because commonly used laboratory animals such as rats express urate oxidase, and kidney damage can occur over time [19]. On day 20, UA levels of S12 treatment group were significantly lower than model group, but BUN and Cr of S12 treatment group and model group were higher than control group. Thus *Lactobacillus* can reduce UA to some extent. These results are consistent with previous reports [20, 21]. The reason is that *lactobacillus* can hydrolyze nucleosides in food and compete with the intestinal epithelium to absorb nucleosides, but does not influence the free nucleosides in food [22]. On day 20, although there was no significant difference in Cr and BUN between S12 treatment groups and model group, model group tended to worsen kidney damage. However, we observed no such toxicity for *Lactobacillus*, suggesting that it provides protection against acute hyperuricemia induced kidney injury [23, 24]. Considering the short duration of this study, long-term observation of probiotic therapy is still needed. Currently, only two studies have reported the effects of probiotics to reduce hyperuricemia in rat model [11, 12]. Our results are consistent with these studies. However, our study developed better hyperuricemia rat model because the rats were administrated with yeast-extract containing high contents of purine and oxonic acid potassium simultaneously to induce hyperuricemia, while in the other two studies the animals were either administrated with oxonic acid potassium alone or administrated with high purine die and oxonic acid potassium consecutively [11, 12]. Although our experimental setup has limitation because we did not employ known effective drug for hyperuricemia as positive control for LAB, our data based on hyperuricemia model may better reveal beneficial effects of LAB for hyperuricemia therapy.

## Conclusion

In summary, we successfully screened *Lactobacillus paracasei* from 18 traditional pickles and demonstrated its ability to degrade nucleosides in vitro and UA in vivo. Rat model of chronic hyperuricemia revealed the potential of *Lactobacillus* to reduce kidney damage. These results suggest that microecological treatment with *Lactobacillus* represents a feasible option for patients with chronic hyperuricemia, especially those with impaired renal function.

## Methods

### Isolation of LAB

Eighteen samples of pickles (pickled Chinese cabbage) were purchased from different parts of China. Each sample (1 g) was blended with 9 mL of pure water and diluted sample was plated onto MRS medium (Difco, USA), and incubated at 37 °C until single colonies grew. Each single colony was preliminarily screened for LAB by gram staining, H_2_O_2_ test and microscopic evaluation (100 × oil). All samples were stored at -80 °C.

### Measurement of inosine and guanosine levels

HPLC was used to detect inosine and guanosine levels simultaneously. 33.7 mg inosine and 35.7 mg guanosine were dissolved in 100 mL K_3_PO_4_ solution (100 mmol/L, pH 7.0) and 20 uL of each solution was injected into a HPLC device (LC-20A, Shinadzu Corporation, China) equipped with a column 5C18-AR-II (4.6 × 250 mm, Cosmosil, China). The final flow phase (0.1 umol/L NaHPO_4,_ 0.187 mol/L H_3_PO_4_) was performed at a flow rate of 1 mL/min, a wavelength of 254 nm, a retention time of 40 mins and a column temperature of 30 °C.

To assess their ability to degrade inosine and guanosine, 1% LAB was inoculated in 10 mL of liquid MRS and cultured for 48 h at 37 °C in oxygen free conditions. MRS solution (2 mL) was then centrifuged at 4000×*g* for 5 mins at 4 °C. The sediment was rinsed with pure water and resuspended in 750 uL of inosine-guanosine solution at 37 °C for 60 min with shaking (120 rpm). The solutions were analyzed by HPLC as described above. The rate of inosine and guanosine degradation by LAB strains was assessed according to the following equation: (v = [(0.9C-X) 0.9C] × 100%, X: the remaining area of ionisine and guanosine on the chromatogram V = speed of degradation (g/L/min).

### Acid tolerance assay

Candidate LAB strains (1%) were inoculated in liquid MRS (Difco, USA), and cultured for 24 h at 37 °C under oxygen free conditions in anaerobic chest with AnaeroPack (Beijing BY Tech, China). Liquid MRS (5%) was then injected into 10 ml MRS and cultured at 37 °C. ODs were assessed at hourly intervals from 0 to 4 h using a spectrophotometer (Nanodrop, Gene Limited, China). Briefly, the number of bacteria in the bacterial suspension per unit volume was measured at OD600. A standard curve was established with the corresponding OD value and bacteria number (OD600 = 1, 2 × 10^9^ cfu/ml).

### Bile tolerance assay

Candidate LAB strains (1%) were inoculated in liquid MRS and cultured for 24 h at 37 °C under oxygen free conditions. Liquid MRS (5%) was then injected into 10 ml of MRS (pH 5.6, 0.1 g/100 mL) and cultured at 37 °C. ODs were assessed at hourly intervals from 0 to 4 h using a spectrophotometer.

### Pepsin and trypsin tolerance assay

Candidate LAB strains (1%) were inoculated in liquid MRS and cultured for 24 h at 37 °C under oxygen free conditions. Liquid MRS (5%) was injected into 10 ml MRS of pH 3, pepsin (0.3 g/100 mL) and of pH 8, trypsin (0.1 g/100 mL), respectively, and cultured at 37 °C. ODs were assessed at hourly intervals from 0 to 4 h.

### Sequencing

16S rDNA sequencing was performed following previously described method [19]. Briefly, DNA was extracted using the CTAB method and DNA concentrations were quantified using a NanoDrop and agarose gel electrophoresis. Illumina Miseq sequencing was used to perform multiple splicing. VSEARCH (v2.4.2) software was used to classify all high-quality sequences with 97% similarity for OTU classification and beta diversity analysis. Finally, an evolutionary tree was built and compared to the Silva database (v123). The microbial communities of the four samples were compared and analyzed according to OTU classification. Since there were multiple OTUs corresponding to the same genus or species, Heatmap was drawn and the TOP30 species in richness ranking were provided.

### Animal model

Animal experiments were carried out at Guangzhou Medical University (Guangzhou, China) following NIH guidelines for the Care and Use of Laboratory Animal and the protocols were approved by Animal Use and Care of Committee of Guangzhou Medical University (Approval No. GMU00327). Male SD rats (42 days old) were provided by Animal Center of the Traditional Chinese Medicine University of Guangzhou, China (SCXK 2013–0034). The rats were housed in a specific pathogen-free (SPF) laboratory at a constant temperature (22 ± 1 °C) and 40–50% humidity, with a 12 h light/dark cycle and free access to chow and water. The rats were randomly divided into three groups (*n* = 10): (1) control; (2) hyperuricemia model; (3) S12 strain treatment. All groups (excluding controls) were administered with yeast extract (20 g/kg body weight) by gavage combined with oxygen oxazine acid potassium (350 mg/100 g) by intraperitoneal injection for 20 days. On the 10th day, S12 strain group was administered 1.0 ml of S12 (1.5 × 10^9^ cfu/ml) by gavage for 10 days. On days 0, 10 and 20, blood was collected and centrifuged at 4000 r/min for 5 mins at 4 °C to obtain serum. Serum uric acid (UA), urea nitrogen (BUN) and creatinine (Cr) levels were then determined using Hitachi 747 automatic analyzer following standard protocols. At the end of experiments, the rats were sacrificed after being intraperitoneally anesthetized with sodium pentobarbital (65 mg/kg) according to the guidelines for euthanasia in the Guide for Care and Use of Laboratory Animals. The kidney tissues were dissected and fixed in 40 g/L paraformaldehyde, wax-embedded and cut into serial sections. The sections were then stained with hematoxylin and eosin (HE), and observed under microscope.

### Statistical analysis

Statistical analysis was performed using SPSS software and GraphPad Prism 5. Data are presented as the mean ± SD. *P*-value < 0.05 was considered statistically significant.

## Data Availability

All data and material are available upon request to correspondence author.

## Abbreviations

Cr: Creatinine
HE: Hematoxylin and eosin
LAB: Lactic acid bacteria
UA: Uric acid
